# SOCIAL ISOLATION AND HOSPITALIZATION IN A NATIONAL SAMPLE OF COMMUNITY-DWELLING OLDER ADULTS BY DEMENTIA STATUS

**DOI:** 10.1093/geroni/igad104.3595

**Published:** 2023-12-21

**Authors:** Mary Louise Pomeroy, Katherine Ornstein, Gilbert Gimm, Thomas Cudjoe

**Affiliations:** Johns Hopkins University, Baltimore, Maryland, United States; Johns Hopkins University, Baltimore, Maryland, United States; George Mason University, Fairfax, Virginia, United States; Johns Hopkins University School of Medicine, Baltimore, Maryland, United States

## Abstract

Social isolation poses risks for poor health, yet its impact on hospitalization for persons with dementia is unknown. Dementia may result in reduced social contact that increases the risk of hospitalization. This observational cohort study examined longitudinal associations between social isolation and hospitalization among a nationally-representative sample of community-dwelling older adults with and without dementia. Using the National Health and Aging Trends Study, we conducted multivariable analyses for 5,533 Medicare beneficiaries. Social isolation was measured using a multi-domain 4-item typology. Logistic regression tested associations between social isolation in 2015 and self-reported hospitalization over the next twelve months, stratifying by dementia status. Covariates included sociodemographics and health characteristics. About 21.5% of older adults were socially isolated, with a much higher proportion of social isolation among those with dementia (38.0%) versus without (20.7%) (p < .001). Overall, the odds of hospitalization were not significantly associated with social isolation (OR 1.13; p = .184). While there was no association between social isolation and hospitalization among those without dementia (OR 1.09; p = .394), social isolation was significantly associated with hospitalization among older adults with dementia (OR 2.07; p = .001). Among older adults with dementia, social isolation increased the predicted probability of hospitalization by about 14.1% over one year (p = 0.030). In this population-based study, social isolation was common among community-dwelling older adults with dementia and significantly associated with increased odds of hospitalization over twelve months. Efforts to reduce hospitalization should explore ways to bolster social relationships for older adults with dementia.

